# Galanin family peptides: Molecular structure, expression and roles in the neuroendocrine axis and in the spinal cord

**DOI:** 10.3389/fendo.2022.1019943

**Published:** 2022-12-06

**Authors:** Sipin Zhu, Xiaoyong Hu, Samuel Bennett, Oscar Charlesworth, Shengnan Qin, Yuliang Mai, Haicheng Dou, Jiake Xu

**Affiliations:** ^1^ Department of Orthopaedics, The Second Affiliated Hospital and Yuying Children’s Hospital of Wenzhou Medical University, Wenzhou, Zhejiang, China; ^2^ Molecular Lab, School of Biomedical Sciences, University of Western Australia, Perth, WA, Australia; ^3^ Guangdong Provincial Key Laboratory of Industrial Surfactant, Guangdong Research Institute of Petrochemical and Fine Chemical Engineering, Guangdong Academy of Sciences, Guangzhou, China

**Keywords:** galanin, galanin-like peptide, alarin, neuroendocrine axis, spinal cord, spinal cord injury

## Abstract

Galanin is a neurohormone as well as a neurotransmitter and plays versatile physiological roles for the neuroendocrine axis, such as regulating food intake, insulin level and somatostatin release. It is expressed in the central nervous system, including hypothalamus, pituitary, and the spinal cord, and colocalises with other neuronal peptides within neurons. Structural analyses reveal that the human galanin precursor is 104 amino acid (aa) residues in length, consisting of a mature galanin peptide (aa 33-62), and galanin message-associated peptide (GMAP; aa 63-104) at the C-terminus. GMAP appears to exhibit distinctive biological effects on anti-fungal activity and the spinal flexor reflex. Galanin-like peptide (GALP) has a similar structure to galanin and acts as a hypothalamic neuropeptide to mediate metabolism and reproduction, food intake, and body weight. Alarin, a differentially spliced variant of GALP, is specifically involved in vasoactive effect in the skin and ganglionic differentiation in neuroblastic tumors. Dysregulation of galanin, GALP and alarin has been implicated in various neuroendocrine conditions such as nociception, Alzheimer’s disease, seizures, eating disorders, alcoholism, diabetes, and spinal cord conditions. Further delineation of the common and distinctive effects and mechanisms of various types of galanin family proteins could facilitate the design of therapeutic approaches for neuroendocrine diseases and spinal cord injury.

## Introduction

Galanin peptide was discovered from the porcine intestinal tract, and was shown to have the potential to affect smooth muscle contraction and glucose metabolism across species (1). The galanin gene, *Gal*, is known to encode a neuroendocrine peptide which is widely expressed in the central and peripheral nervous systems, gastrointestinal tract, pancreas, adrenal gland, and urogenital tract for the regulation of various biological functions (2–7). The encoded precursor, preprogalanin (PPGAL), is proteolytically processed into the mature galanin and galanin message-associated peptide (GMAP) (8). Human galanin is a neuromodulator and appears to function in regulatory roles for nociception, synaptic neurotransmission, and neural activities including cognition, mood, epileptic activity, and spinal reflexes (9–12). Mutation analyses of human *GAL* gene reveal that it is associated with autosomal dominant epilepsy in humans (13), a condition which features recurrent seizures of the temporal lobe and dysfunction of the auditory system. In line with this finding, mice with *Gal* deficiency showed defects in nociceptive functions after peripheral nerve injury (11), whereas galanin-treated rats and *Gal-*overexpressing transgenic mice exhibit impairment in trace cued fear conditions (12). Galanin peptide also displays versatile physiological roles in metabolic processes, mediating insulin metabolism, somatostatin release, and intestinal smooth muscle activity (14, 15).

GMAP is derived from the C-terminal region of the galanin precursor. Whilst the role of galanin is well established, the role of GMAP is relatively less characterized. GMAP mediates spinal flexor reflex in rats (16, 17) and to exhibit antifungal activity of innate immune cells and tissues (18, 19). Recombinant GMAP was able to inhibit forskolin-stimulated adenylate cyclase activity in the spinal cord (20), it remains to be determined how GMAP exerts common or distinctive pharmacological actions as compared to galanin peptide.

Additionally, the galanin peptide family includes galanin-like peptide (GALP) and alarin, which was discovered in tumors derived from neuroblast tissues (21, 22). GALP appears to have a variety of physiological functions, whilst alarin seems to be a vasoactive peptide (23, 24). GALP is a similarly structural protein to galanin, which appears to regulate postsynaptic currents (25), and to mediate food intake, sexual behaviours, anorectic action, and body weight (26–28). Alarin, a differentially spliced variant of GALP, is implicated in the ganglionic differentiation of neuroblastic tumors and vasoactive effects in the skin (23), and has been shown to have anti-bacterial effects (29).

The pleiotropic functions of the galanin peptide family suggest that they act as homeostatic signaling molecules of intercellular communication in the neuroendocrine axis. Recent research provides contemporary insights into the functions of galanin, GMAP, GALP and alarin *via* binding of galanin receptors (GALR), which consist of GALR1, GALR2, and GALR3 (30, 31). It is likely that galanin, GMAP, GALP and alarin may selectively bind different GALRs in various tissues to regulate their functions *via* G protein coupled receptor - mediated intracellular molecular mechanisms, such as second messengers (30, 31). In this review, we survey the molecular structure, expression, and roles of the galanin peptide family in the neuroendocrine axis and spinal cord. Further understanding of their unique and differential functions is critically important to the development of these molecules as potential therapeutic targets for the treatment, prevention, and cure of related diseases and disorders.

## Molecular structural analysis and expression of galanin

**Figure 1:**
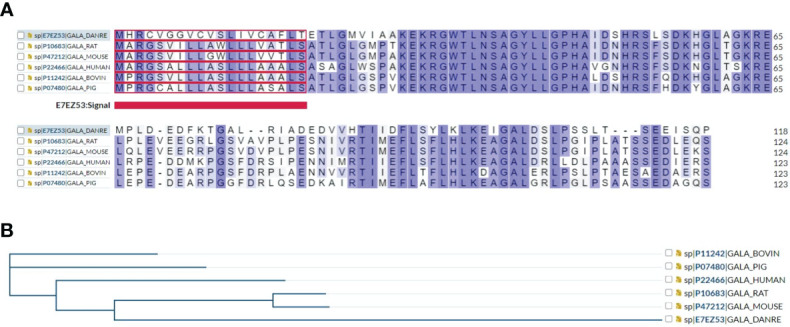
**(A)** Multiple sequence alignment results show that human galanin shares approximately 70% amino acid sequence identity and similarity with other galanin family proteins including rat, mouse, zebrafish, bovine, and pig. **(B)** A phylogenetic tree of galanin family proteins is presented using https://www.uniprot.org/align.

Multiple sequence alignment showed that human galanin shares approximately 70% amino acid sequence similarity and identity to rat, mouse, bovine, and pig and zebrafish, indicating a conserved structural and functional relationship among species (**Figure 1A**), and a common phylogenetic tree (**Figure 1B**).

**Figure 2:**
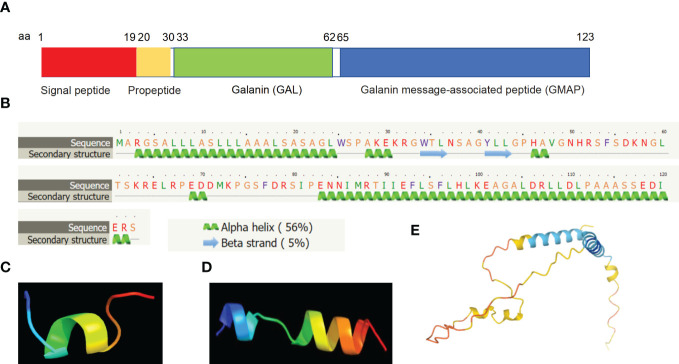
Molecular structural analyses of galanin. **(A)** Human galanin is comprised of a signal peptide of 19 amino acid residues (aa 1-19), a propeptide (aa 20-30), mature galanin peptide of 30 amino acid residues (aa 33-62) and the C terminal fragment, GMAP (aa 59 -123). **(B)** Secondary structure predicts characteristics of five alpha-helices and two beta-sheets based on bioinformatic analysis. **(C, D)** 3D structure analysis showing that mature galanin was predicted to resemble transportan **(C)**, and GMAP was predicted based on the bag family molecular chaperone regulator 5 **(D)** using the Phyre2 web portal. **(E)** 3D structure analysis result of the full length of galanin is shown using the Alphafold web portal (https://alphafold.ebi.ac.uk/).

Molecular structure analyses revealed that mature human galanin is a secreted peptide of the galanin family which consists of a peptide of 30 amino acid residues in length. The full sequence of *GAL* encodes PPGAL, which is a 123 amino acid peptide containing a signal sequence from amino acid residues 1-19 and the galanin precursor peptide of 104 amino acids (aa 20-123) and is predicted to be approximately 12 kDa in size (**Figure 2A**). The galanin precursor is cleaved to yield two peptides, a mature galanin peptide consisting of 30 amino acid residues (aa 33-62) and the C-terminal GMAP peptide from amino acid residues (aa 59 -123) (**Figure 2A**). The GMAP sequence also consists of a phosphorylated serine residue at amino acid residue 117. Secondary structure analyses revealed that mature galanin contains five alpha helices and two beta strands by using the web-based Phyre2 portal (32) (**Figure 2B**). 3D structure analysis of mature galanin was predicted to resemble transportan (**Figure 2C**), and GMAP (aa 59 -123) was predicted to resemble the bag family molecular chaperone regulator 5 (**Figure 2D**) by using web-based Phyre2 portal (32). Further, the full length of galanin is predicted using the Alphafold web-portal (33), and is shown to be consistent with its secondary structure (**Figure 2E**).

Early studies indicated galanin expression to be concentrated in the neurohypophysis, hypothalamus, and sacral spinal cord regions by using radioimmunoassay and immunocytochemistry (34). Further transcriptomic analyses reveal that human *GAL* is widely expressed in the central nervous system (CNS) and gastrointestinal tract tissues, such as appendix (35). *Galanin* gene expression appears to be upregulated by nerve injury and lesions of the CNS, and to have a neuromodulatory anticonvulsant effect on hippocampal excitability (36–40). Galanin seems to affect cognitive functions, including memory and attention, and attenuated *GAL* expression is a potential therapeutic implication for neurocognitive diseases, such as Alzheimer’s disease (41, 42). Interestingly, *Gal* mRNA expression during embryonic development in rats indicates its involvement in the formation of several sensory systems, including the nervous and skeletal systems (43), and galanin secretion by the anterior pituitary is postulated to be upregulated by circulating estrogen levels (44), indicating its pleiotropic roles.

**Figure 3:**
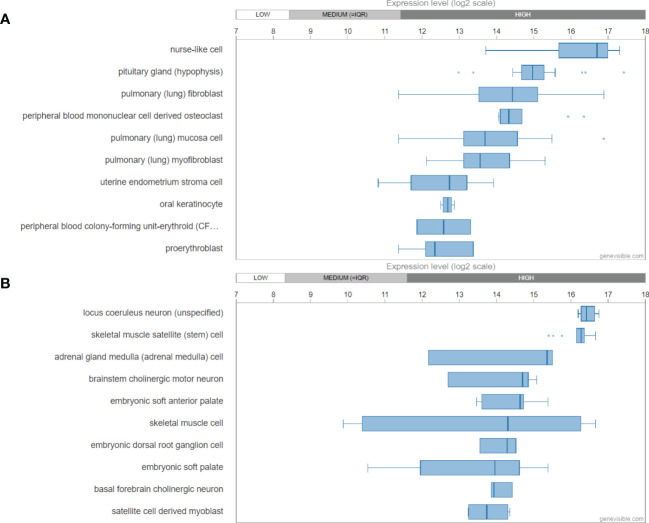
mRNA expression profiling of galanin gene in both human **(A)** and mouse **(B)** tissues, showing the then most highly galanin expressing tissues. These data were predicted by using bioinformatics based on the Genevisible^®^ (http://genevisible.com).

Utilizing Genevisible^®^ (45), human *GAL* mRNA was found most abundantly expressed in nurse like cells, pituitary glands, and pulmonary fibroblasts (**Figure 3A**). In mouse tissues, *Gal* mRNA was revealed most highly expressed in locus coeruleus neurons, skeletal muscle satellite cells, adrenal gland medulla, and brainstem cholinergic motor neurons (**Figure 3B**).

## Molecular structural analysis and expression of GALP and alarin

**Figure 4:**
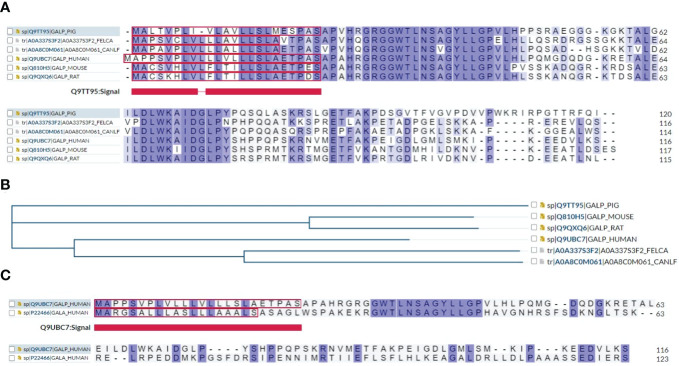
**(A)** Multiple sequence alignment results show that human GALP shares sequence identity or similarity to mouse, rat, pig, dog and cat. **(B)** A phylogenetic tree of GALP family proteins is presented using https://www.uniprot.org/align. **(C)** Multiple sequence alignment results show that GALP shares about 50% of sequence homology with galanin.

Multiple sequence alignment showed that human GALP shares sequence similarity and identity with mouse, rat, pig, dog, and cat, indicating a very conserved structural and functional relationship among species (**Figure 4A**) and a common phylogenetic tree (**Figure 4B**). In addition, multiple sequence alignment showed that human GALP shares amino acid sequence similarity and identity to galanin, suggesting they belong to the same family members of galanin (**Figure 4C**). Further, alarin is a differentially spliced form of *GALP* gene which encodes a 25 amino acid neuropeptide (GenBank accession no. DQ155644) (23).

**Figure 5:**
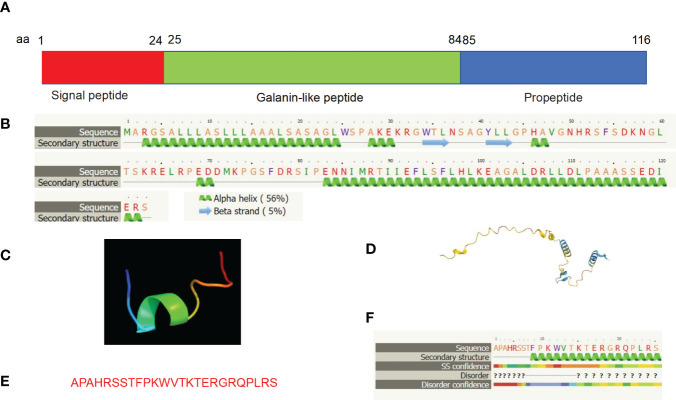
Molecular structural analyses of GALP. **(A)** Human GALP is comprised of a signal peptide of 24 amino acid residues (aa 1-24), Galanin-like peptide (aa 25-84) and a propeptide at the C terminus (aa 85 -116). **(B)** Secondary structure predicts characteristics of five alpha-helices and two beta-sheets based on bioinformatic analysis using the Phyre2 web portal. **(C)** 3D structure analysis of Galanin-like peptide was predicted to resemble transportan, same as that of galanin. **(D)** 3D structure analysis result of the full length of GALP is shown using the Alphafold web portal (https://alphafold.ebi.ac.uk/). **(E)** Alarin is a differentially spliced form of *GALP* gene which encodes a 25 amino acid neuropeptide (GenBank accession no. DQ155644). **(F)** Secondary structure of alarin based on bioinformatic analysis using the Phyre2 web portal.

Molecular structure analyses reveal that mature human GALP is a secreted peptide, consisting of a signal sequence from amino acid residues 1-24, a mature GALP from amino acid residues 25-84, and propeptide from amino acid residues 85-116 (**Figure 5A**). Secondary structure analyses reveal that GALP consists of five alpha helices and two beta strands by using the web-based Phyre2 portal (32) (**Figure 5B**). 3D structure analysis of GALP (amino acid residues 25-84) was predicted to resemble transportan (**Figure 5C**) by the Phyre2 web portal (32), which is the same as that of galanin. Further, the full length of GALP is also predicted based on the Alphafold web portal (33), and is shown to display consistent features with its secondary structure (**Figure 5D**). Further, alarin is a differentially spliced form of GALP gene which encodes a 25 amino acid neuropeptide (GenBank accession no. DQ155644) (23). (**Figures 5E, 5F**).

GALP mRNA was found expressed in the hypothalamus, pituitary gland and guts (21, 46, 47), and upregulated by osmotic stimulation (48). GALP was also found to be regulated by leptin (46) and by orexin (49), suggesting its role in the modulation of neural activities.

**Figure 6:**
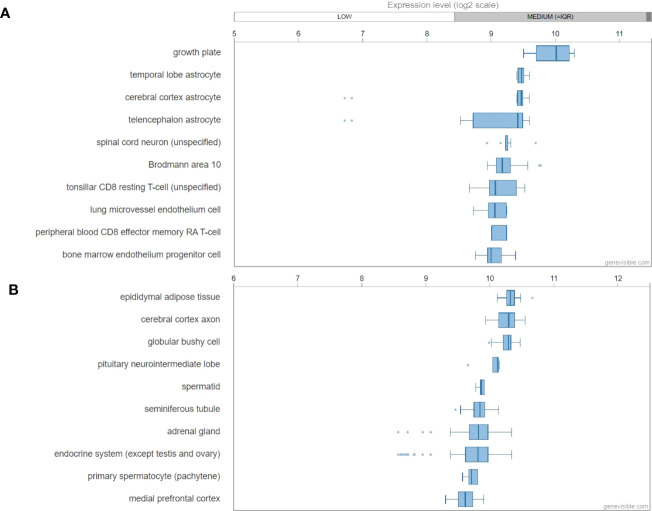
mRNA expression profiling of GALP gene in both humans **(A)** and mouse **(B)** tissues, showing the ten most highly galanin expressing tissues. These data were predicted by using bioinformatics based on the Genevisible^®^ (http://genevisible.com).

By the analyses of Genevisible^®^ (45), human *GALP* mRNA was most abundantly expressed in growth plate, temporal lobe astrocyte, cerebral cortex astrocyte, telencephalon astrocyte and spinal cord neuron (**Figure 6A**). In rat tissues, *Galp* mRNA was most highly expressed in epididymal adipose tissue, cerebral cortex axon, globular bushy cell, pituitary neurointermediate lobe, and spermatid (**Figure 6B**).

## The role of galanin, GALP and alarin in the neuroendocrine axis

The widespread expression and physiologic functions of galanin in the central and peripheral nervous systems, and in tissues, such as the gastrointestinal tract and pancreas, demonstrate its importance and versatility for neuro-hormonal homeostasis (50). Galanin has been suggested to play significant roles in the neuroendocrine axis (9, 10, 51) for the mediation of diverse neuronal pathological conditions of ageing (51, 52), feeding and energy metabolism (28, 53), pancreatic secretion of insulin and diabetes (54, 55), mood and behaviour (56, 57), seizure (13, 36), pain experience (53), bone density (58), cartilage growth plate physiology and repair (59) and skin (60).

In the endocrine system, galanin was able to inhibit glucose-induced insulin release (61–63), by suppressing glucose-induced insulin secretion from rat islet cells (63). Accordingly, galanin has been implicated in regulating glucose metabolism, by improving insulin resistance and diabetes mellitus type 2 (54, 64).

In the nervous system, galanin has been suggested to regulate acetylcholine release (65, 66), and glutamate release (67) from the hippocampus, by acting as an inhibitory neuromodulator. Further, galanin was found to suppress the release of acetylcholine, suggesting a role for galanin in the cholinergic regulation of Alzheimer’s disease (68–70). Galanin was also involved in addiction to alcohol (71), and in population-dependent vulnerability to alcoholism (72). At the cellular level, galanin fibres were detected in the sympathetic nerves of the basal forebrain of Alzheimer’s disease patients (73). Consistently, galanin was shown to attenuate Abeta-induced caspases-3 and -9 cleavage, and thus protect against Abeta-mediated neuron toxicity in rat cholinergic basal forebrain (73, 74). Interestingly, *galanin* mRNA expression was found to be upregulated in central and peripheral nerves following axotomy (37, 38, 75), which is suggestive of a role in the regulation of neural regeneration (75–77).

In the skeletal system, galanin was found to modulate the mechanosensitivity of fine afferent nerve fibres of the rat knee joint (78). Additional study showed that galanin-like immunoreactivity was detected in cortical bone, periosteum, endosteum, and surrounding skeletal muscle of normal rib at a minimum level, whereas galanin was abundantly detected in chondrocytes of the hypertrophic zone and in the reserve zone in costal cartilage plates, but not in chondrocytes in the proliferative zone of costal cartilage or skeletal muscle fibers (59). In comparison, a rodent model of rib-fracture found that galanin expression was increased in callus tissues with staining detected in cells (osteoprogenitor cells, osteoblasts, and chondrocytes) and in tissue matrices (cartilaginous, osseous, and periosteal) during bone healing; whilst serum galanin concentrations were consistently found to be increased at 2 weeks following fracture (59). In line with these findings, galanin-containing nerve fibres were observed in bone of rats from gestational day 16 to postnatal day 21 at various levels, ranging from the epiphyseal perichondria to the diaphyseal periosteum at gestational day 16; the bone marrow cavity as well as the inter-condylar eminence of the knee joint at postnatal day 1; the cartilage canals of both epiphyses at postnatal day 7; the secondary ossification centres at postnatal day 10; the bone marrow of both epiphyses at postnatal day 14; suggesting their sensory origin in various regions during skeletal development (79). In a mouse calvarial injection model, galanin treatment was found to facilitate bone formation associated with injury *via* the inhibition of cytokine production, such as excess TNF-alpha and IL-1beta, after bone injury (80). Collectively, these studies highlight the important roles of galanin in skeletal biology and diseases.

GALP shares similar structure and effects on postsynaptic currents as galanin (25). However, GALP also exhibits a unique role in regulating the intrinsic membrane properties when compared to galanin for the modulation of neurons in the arcuate nucleus, suggesting that galanin and GALP might regulate energy balance and reproductive function in a different way (25). As a neuropeptide expressed in the arcuate nucleus of the posterior pituitary gland and in the hypothalamus (47, 81, 82), GALP is involved in the regulation of appetite and possibly has additional roles, such as in inflammation (81, 83), metabolism and reproduction (84, 85), sex behaviour of adult male rats (26), as well as stress involved in the hypothalamo-neurohypophyseal system (82). However, the mechanism by which galanin or GALP govern common and differential activities remains to be elucidated.

GALP appears to be a hypothalamic neuropeptide which acts through GALR (47, 86–88), and has diverse roles in the central nervous system, including the regulation of luteinizing hormone (LH) (89, 90), metabolism and reproduction, food intake, and body weight (90, 91). Alarin is a splice variant of GALP encoding a short peptide, APAHRSSTFPKWVTKTERGRQPLRS (22, 23) and has been shown to exhibit vasoconstriction and anti-edema properties in the microvasculature of skin (23), which is distinctive from GALP and might function independently of GALR. Alarin was also found to have anti-bacterial effects on gram-negative bacteria by causing bacterial membrane blebbing (29). In addition, alarin was able to stimulate food intake and LH secretion in rodent studies (92). In line with these features, alarin was mostly expressed in ganglia of ganglioneuroma and ganglioneuroblastoma, differentiated tumor cells of neuroblastoma tissues, but not in undifferentiated neuroblasts (22). The distribution of alarin-like immunoreactivity was identified in the adult murine brain with dissimilar expression pattern to that of GALP (93), suggesting possible distinct functions between GALP and alarin in the regulation of reproduction and metabolism.

In addition, recent studies have shown the involvement of galanin in brainstem cardio-respiratory function. For instance, galanin is notably present within a subset of neurons of a respiratory central chemoreceptor region in the rostral ventrolateral medulla, namely the retrotrapezoid nucleus (RTN) in the parafacial region in neonate and adult rodents (94–97). These neurons could be characterized by Phox2b-positive and VGlut2-positive and thus were excitatory, although galanin itself induced inhibitory effects on respiratory activity (94, 98).

## The emerging role of galanin, GALP and alarin in the spinal cord

In the spinal cord system, galanin was detected in sensory neurons at the dorsal horn of the spinal cord, and has been implicated in mediating nervous impulses and neuropathic pain caused by peripheral nerve injury (99). Galanin-immunoreactive nerve fibers and bladder end-organ nerve supply were reduced after spinal cord injury (SCI) (100). Consistently, galanin expression in nerve fibers, L1 segment, and in the urinary bladder detrusor and urothelium was decreased after SCI (101). In contrast, the expression of galanin was increased in localisations of the gray matter of the rostral lumbar, and lumbosacral spinal cord, including regions of the S1 spinal cord, dorsal commissure in the L4 segment, and in dorsal root ganglia (DRG L1, L2, L6, and S1), which was accompanied with an increase in brain-derived neurotrophic factor (BDNF) expression in these areas after SCI. These results suggest that galanin expression associated with urinary bladder dysfunction might be a consequence of SCI due to changes in the spinal micturition reflex and bladder hyperreflexia (101). Physical activity combined with hormone treatment has been shown to increase the gene expression of galanin and BDNF following SCI, which could be an important therapeutic outcome to attenuate secondary neuronal degeneration and alleviate pain in SCI (102, 103). By using total RNAseq, we have recently found that *Gal* mRNA was up regulated at day 1, and returned to normal level at day 21 after SCI in rats (our unpublished observations), indicating a time-dependent regulation of *Gal* expression following SCI. This finding requires further research for optimal timing of therapeutic interventions for SCI involving galanin. Importantly, galaninergic neurons were frequently located between L2 and L5 segments of the medial lamina VII, with a maximal density within L4, which would appear to contain the spinal ejaculation generator (104–106), and to have potential treatment implications for SCI patients. Further research is therefore required to develop possible therapeutic strategies of SCI involving galanin.

Additional research supports the involvement of galanin in nociceptive sensory processing of the spinal cord (11, 107). For example, *Gal* mutant mice showed higher sensitivity to threshold noxious stimuli than wild-type controls in the absence of peripheral nerve injury; whereas spontaneous and evoked neuropathic pain behaviours were found to be compromised in mutant mice following peripheral nerve injury, indicating that galanin possibly plays a role in the regulation of inhibitory and excitatory nociceptive processing of the spinal cord (11). Consistently, galanin was found to lower spinal excitability and the firing of locus coeruleus neurons (107). It was further revealed that antinociception of galanin in dorsal root ganglia neurones following nerve injury is related to the stimulation of galanin receptor 1 (GALR1) on dorsal horn neurones, whereas the pro-nociceptive effect of galanin appears to be related to presynaptic GALR2 on primary afferents (108), indicating that galanin might interact with selective galanin receptors to regulate neuropathic pain, and that a selective GALR1 agonist could be an effective treatment approach.

The role of GMAP in the spinal cord appears less clear. Studies have shown that spinal flexor reflex is possibly mediated by variation of GMAP receptor subtypes and segments of GMAP sequence (16, 17). The role of GALP or alarin in SCI remains unknown and GALP is weakly expressed in spinal cord or SCI samples according to total RNAseq data (our unpublished observations).

Further studies are required to define the molecular mechanisms and cellular signaling pathways of galanin and GALP in neuropathic pain, healing, repair and regeneration following SCI and related disorders. These studies will increase our understanding of the pathological processes critically involving galanin family peptides in spinal cord injury, dysfunction, and pain. This will ultimately lead to the development of novel and improved treatments for SCI patients.

## Summary

**Figure 7:**
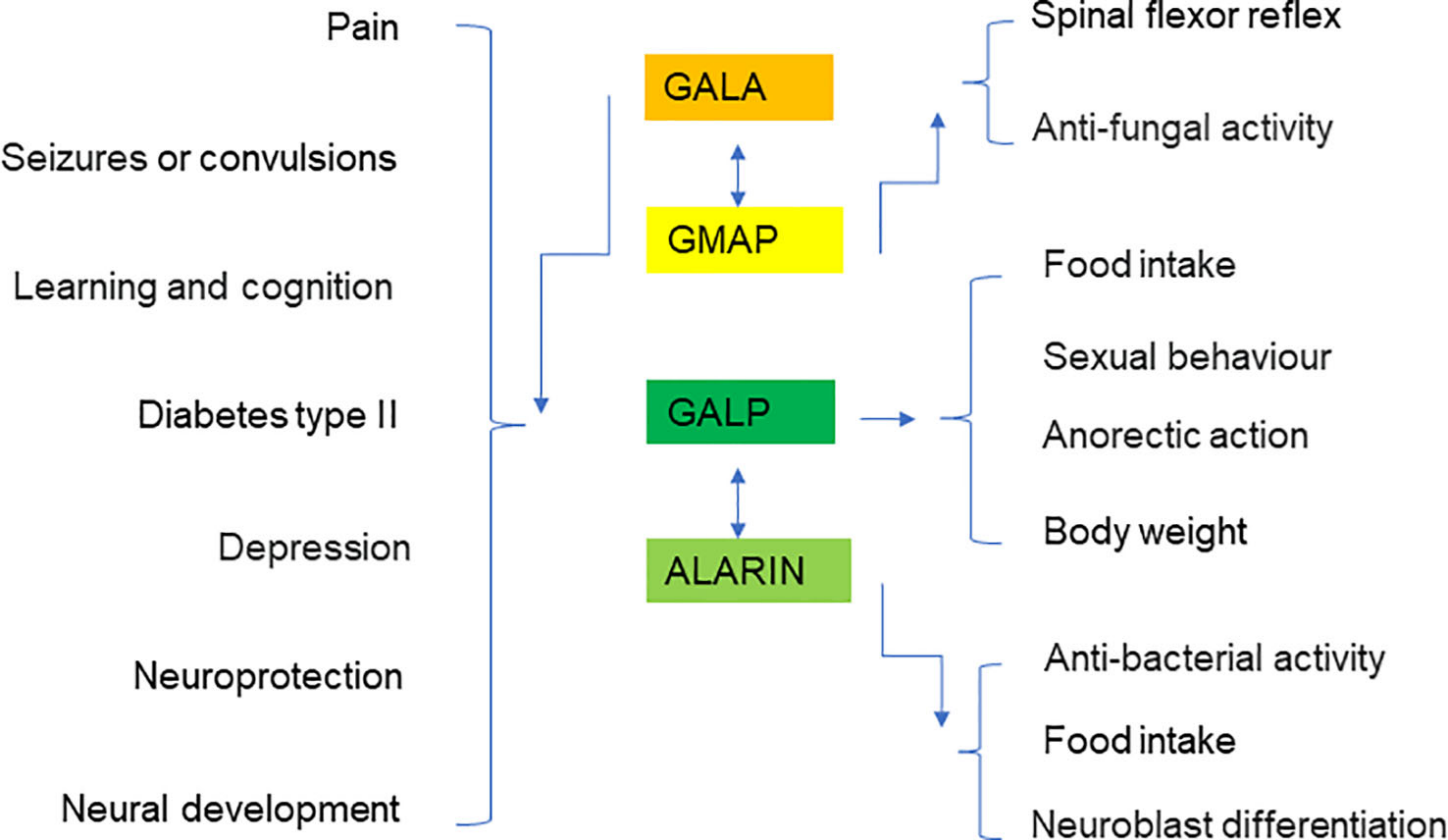
Summary diagram showing the putative roles of galanin, GMAP, GALP, and alarin in various organs and disease conditions.

Galanin is a neuropeptide that is expressed in a wide range of tissues including the brain, spinal cord, and gut. It plays a crucial role in mediating the neuroendocrine axis, and has been implicated in many physiological functions including neuroprotective activity, neurogenesis, nociception regulation, cognitive functions, feeding, and mood status (**Figure 7**). Dysregulation of galanin is associated with pathological conditions including Alzheimer’s disease, seizures, eating disorders and addiction. GALP is a hypothalamic neuropeptide and shares similar structure and effects on postsynaptic currents as galanin. Both galanin and GALP appear to play vital roles in the spinal cord system. However, the exact mechanisms by which the galanin family proteins exert their pleiotropic roles remain to be fully illuminated. It is likely that galanin and GALP selectively bind their tissue-specific receptors for the regulation of various biological functions, and in associated disease conditions. Further defining the molecular mechanisms and cellular signaling pathways of galanin, GMAP, GALP, and alarin is critically important for the development of improved therapeutic interventions to target this intriguing and efficacious peptide family.

## Author contributions

SZ drafted the manuscript. XH, SB, OC, HD, and SQ contributed to figure formulation and literature collection. SB and JX revised the manuscript. YM and JX contributed to evaluation and assistance in the process of manuscript preparation and supervised the study.
